# Imaging distinct neuronal populations with a dual channel miniscope

**DOI:** 10.3389/fnins.2024.1445020

**Published:** 2024-12-09

**Authors:** Giovanni Barbera, Rashmi Thapa, Navin Adhikari, Yun Li, Da-Ting Lin

**Affiliations:** ^1^Intramural Research Program, National Institute on Drug Abuse National Institutes of Health, Baltimore, MD, United States; ^2^Department of Zoology and Physiology, University of Wyoming, Laramie, WY, United States; ^3^The Solomon H. Snyder Department of Neuroscience, Johns Hopkins University School of Medicine, Baltimore, MD, United States

**Keywords:** miniscope, calcium imaging, dual color imaging, miniature fluorescence microscope, liquid lens, EWL, single-photon, open source

## Abstract

Miniature fluorescence microscopes (miniscopes) are one of the most powerful and versatile tools for recording large scale neural activity in freely moving rodents with single cell resolution. Recent advances in the design of genetically encoded calcium indicators (GECIs) allow to target distinct neuronal populations with non-overlapping emission spectral profiles. However, conventional miniscopes are limited to a single excitation, single focal plane imaging, which does not allow to compensate for chromatic aberration and image from two spectrally distinct calcium indicators. In this paper we present an open-source dual channel miniscope capable of simultaneous imaging of genetically or functionally distinct neuronal populations. Chromatic aberrations are corrected using an electrowetting lens (EWL), which allows fast focal plane change between frames. To demonstrate the capabilities of the dual channel miniscope, we labeled layer specific excitatory neurons or inhibitory interneurons in the medial prefrontal cortex (mPFC) with a red fluorescence protein, and simultaneously imaged neural activity of distinct neuronal populations of freely moving mice via a green GECI.

## 1 Introduction

Understanding the causal relationship between neural network activity patterns and behavior is a long standing, fundamental question in Neuroscience. To better understand how neuronal ensembles regulate different behaviors, calcium imaging with fluorescent indicators has provided unprecedented insights into single cell activity for hundreds to thousands of neurons across timescales of weeks to months. More recently, the miniaturization of fluorescence microscopes (Flusberg et al., 2008; Ghosh et al., 2011) has substantially expanded the scope of behavioral paradigms to which calcium imaging can be applied, ranging from learning and memory (Ghandour et al., 2019; Ziv et al., 2013) to complex behaviors such as social interaction (Li et al., 2017; Liang et al., 2018; Murugan et al., 2017), fear conditioning (Alfieri et al., 2022; Grewe et al., 2017) and operant behaviors (Cameron et al., 2019; Grewe et al., 2017; Siciliano and Tye, 2019; Zhang Y. et al., 2021).

More recent advances in miniaturized fluorescence microscopy include simultaneous imaging and optogenetic manipulation using a dual LED miniscope (Stamatakis et al., 2018; Zhang J. et al., 2021), and preliminary versions of dual-color miniscope are currently being proposed both in commercial and academic systems (Guo et al., 2021; North et al., 2023; Stamatakis et al., 2021).

One way to provide multiple light sources for dual-color imaging or simultaneous imaging and optogenetic manipulation, is to couple external light sources with the miniscope through fiber optic (Srinivasan et al., 2019). This approach, however, does not offer a solution for chromatic aberration for imaging with gradient-index (GRIN) lenses (Phillips et al., 2023), and it could limit animal mobility.

In this paper, we propose a dual-color version of the miniscope capable of compensating for chromatic aberration by alternating both light source and focal plane in interleaving frames.

## 2 Materials and methods

### 2.1 Miniscope

**Figure 1:**
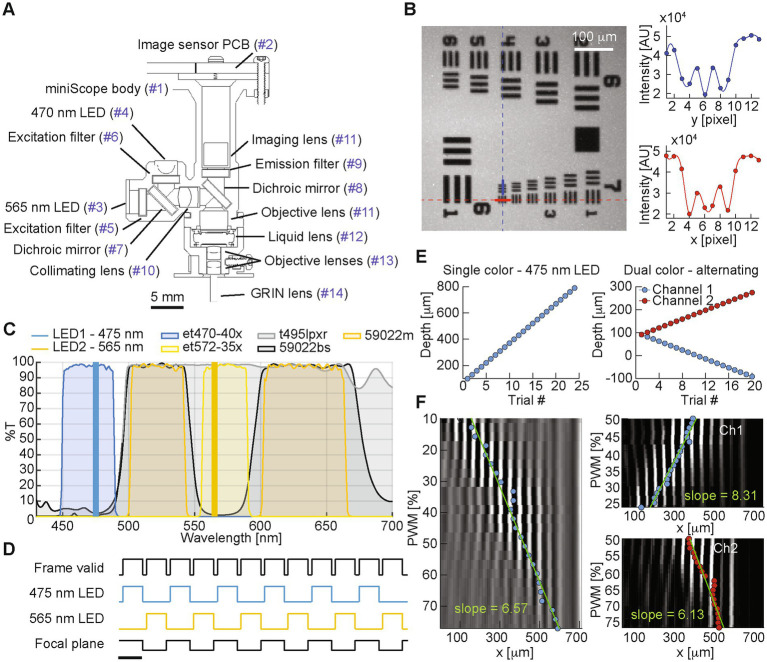
**(A)** Miniscope design (part list matched in Table 1). **(B)** Left: image of a 1951 USAF target. Right: pixel intensity values for the column and row highlighted on the left panel in blue and red, respectively, covering group 7, element 6 with a 4.4 mm resolution and ~3.3× magnification. **(C)** Spectra for the miniscope LEDs and filters. **(D)** Plot showing the timing of miniscope signals in alternating channels configuration, where two interleaved video streams are generated. Scale bar is 100 ms reflecting a representative 10 fps image sensor frame rate. **(E)** Focal plane depth for the different test trials recorded during the imaging of the 45° depth of field target in single channel recording (left), and dual-color alternating configuration (right). Focal plane depth is calculated from the EWL control signal based on the fitted linear model shown in **F**. **(F)** Column-average of the average frame imaging the 15 lp/mm vertical lines from the 45° depth of field target at different focal depths, for single channel recording (left), and dual-color alternating configuration (right).

The miniscope design consists of a dual-LED, single photon fluorescence microscope with tunable lens (A-16F0-P12, Corning Varioptic), designed in Solidworks and 3D-printed in black resin (Protolabs, Maple Plain, MN). The design is similar to Barbera et al. (2022), with the introduction of two independent, spectrally distinct light sources at 470 nm and 565 nm, respectively. The miniscope design is shown in Figure 1A, and the complete list of components is reported on Table 1. A custom PCB hosts the CMOS image sensor (MT9V022, Aptina/Onsemi), which is synchronized to the LEDs and streams data to a custom FPGA control board (Zhang et al., 2018).

**Table 1:** Bill of materials.

#	Manufacturer	Manufacturer P/N	Description	Price	Quantity
#1	Protolabs	Custom miniscope body	Black resin 25 mm tolerance	$122.91	1
#2	Sierra Circuits	Custom image sensor PCB	PCB hosting miniscope images sensor/LED control	$97.14	1
#3	Mouser	997-LXZ1PX01	Luxeon Rebel 565 nm LED	$4.86	1
#4	Mouser	941-XPEBBLL10000302	Cree high power 470 nm LED	$2.16	1
#5	Chroma	ET572/35x	Excitation filter 555–590 nm, 3 × 3 × 1 mm	$12.50	1
#6	Chroma	ET470/40x	Excitation filter 450–490 nm, 3 × 3 × 1 mm	$12.50	1
#7	Chroma	T495lpxr	Main dichroic mirror 5 × 5 × 1 mm	$12.50	1
#8	Chroma	59022 bs	Dichroic mirror 5 × 5 × 1 mm	$12.50	1
#9	Chroma	5922 m	Emission filter 3 × 3 × 1 mm	$12.50	1
#10	Edmund Optics	49271	3 mm Dia. x 6 mm FL, VIS 0° coated, achrom lens	$98.50	1
#11	Edmund Optics	63704	4 mm Dia. × 12 mm FL, VIS 0° coated achrom lens	$90.00	2
#12	Edmund Optics	A-16F0-P12	A-16F0 with FPC-A-12 variable focus liquid lens	$103.50	1
#13	Edmund Optics	84126	2 mm Dia. × 6 mm FL, VIS 0° coated achrom lens	$91.00	2
#14	GRINTech	NEM-100-25-10-860-S-0.5p	1 mm Dia. GRIN lens	$100.00	1

### 2.2 Animals

All experimental procedures were approved by the Institutional Animal Care and Use Committee of the University of Wyoming and the National Institute on Drug Abuse (NIDA) and were conducted in accordance with the guidelines of the National Institutes of Health. Gad2-IRES-Cre male and female breeders were obtained from the Jackson Laboratory (stain # 010802). *Tardbp flox/flox* (*Tardbp^F/F^*) and CaMKII-IRES-Cre; Tardbp^F/F^ male and female breeders were obtained from Dr. Philip Wong’s Laboratory at the Johns Hopkins University School of Medicine. These mouse lines were bred and maintained at the University of Wyoming. Mice were housed up to five mice per cage under a 12 h light/dark cycle and given *ad libitum* access to food and water.

### 2.3 Stereotaxic injection

*Tardbp^F/F^* male and female mice (9–11 months old) were bilaterally injected with viruses at the medial prefrontal cortex (mPFC) region according to the previously published protocol (Thapa et al., 2021; Zhang et al., 2018). Mice were anesthetized and secured in a stereotaxic apparatus, maintaining anesthesia at 1.5% isoflurane in oxygen, and a constant temperature of 35°C with a heating pad. A small hole was drilled on the skull with the help of 0.6 mm dental drill burr and 500 mL of virus was injected bilaterally at the coordinates (A/P: +1.94 mm, M/L: 0.5 mm and D/V: 1.75 mm) with a microliter syringe controlled by a micropump at the flow rate of 500 mL/min. The left mPFC received AAVretro-hSyn-mCherry (1.0 × 10^13^ GC/mL). In the right mPFC, mice were injected with 1:2 diluted AAV1-CaMKII-GCaMP6f (1.3 × 10^13^ GC/mL). The mice were fed with drinking water containing Ibuprofen for 3 days following surgery and allowed to recover for 14 days.

Gad2-IRES-Cre mice (HOM/HET, 12–13 months old) were sequentially labeled with two consecutive viral injections at the right mPFC. The first injection was made with 1:2 diluted AAV1-hSyn-GCaMP6f (1.3 × 10^13^ GC/mL) in the right mPFC to label all neurons with green fluorescence calcium indicator. After 14 days of recovery, a second viral injection was performed at the same region with AAV1-CAG-DIO-NLS-tdTomato (1.0 × 10^13^ GC/mL) to label the GABAergic neurons with tdTomato.

*CaMKII-IRES-Cre; Tardbp^F/F^* mice (3–5 months old) were injected unilaterally with a 1:1 viral mixture of AAV1-CaMKII-GCaMP6f and AAV1-CAG-DIO-NLS-tdTomato such that the GCaMP6f labelled all the pyramidal neurons and tdTomato labelled all the Cre expressing cells. The expression of tdTomato will only occur after the activity of Cre-recombinase has been induced with tamoxifen.

### 2.4 Gradient-index lens implantation

GRIN lens implantation at the right mPFC region was performed according to the previously published lab protocol (Thapa et al., 2021; Zhang et al., 2018). The mice were anesthetized with ketamine/xylazine mixture (ketamine: 100 mg/kg; xylazine: 15 mg/kg) and shaved in the surgical site. After fixing the mouse head in a stereotaxic stage, a triangular skin region was excised, and the remaining skin region was attached to the skull with cyanoacrylate. Using a 1.2 mm drill burr, a hole was drilled at the coordinates (A/P: 1.94 mm, M/L: 0.8 mm) from the bregma. The brain tissue was aspirated layer by layer down to the depth of 1.8 mm using a 27G blunt end needle which was connected to a vacuum system. The needle was attached to a robotic arm at an angle of 10° which was operated using a custom-built software.^1^ The brain was continuously irrigated with artificial cerebrospinal fluid which was bubbled with 95% O_2_ and 5% CO_2_. After the well was clean, a GRIN lens (1 mm diameter) was inserted into the well. The GRIN lens was fixed in its place with two layers of dental cement (Metabond followed by Duralay) and the lens was later covered with custom-built cap. The mice were fed with Ibuprofen through drinking water for 3 days and allowed to recover for a month.

### 2.5 Base mounting

The mouse was anesthetized with isoflurane and fixed in a stereotaxic stage. The dual-color miniscope fitted with a base was brought in close proximity to the lens with the help of a custom-built motorized controller such that the miniscope focused onto the field of view within the lens. The base was affixed in its position with two rounds of dental cement application. After the cement had solidified, the miniscope body was withdrawn and the lens was protected with a cap that fitted in the base.

### 2.6 Repeated *in vivo* calcium imaging

On days where calcium imaging was performed, the mouse was briefly anesthetized using isoflurane. The dual color miniscope was fastened onto the base and the mouse was allowed to recover from isoflurane for 30 min. During recording, the mouse was allowed to explore an open square arena (42 cm × 42 cm × 30 cm) for 15 min (open field test). Calcium images were recorded using NeuView, a custom-built calcium imaging software (Zhang et al., 2018). Optimal focal plane was found by maximizing the number of detected neurons, and the LED power was set low enough to avoid pixel saturation from the image sensor due to peak activity fluorescence. For the dual color recording, LED1 was used to capture the green channel which represents GCaMP6f fluctuations whereas LED2 was used to capture the stationary red signal. The behavior of the mouse in open area was recorded using a behavior recording software (FlyCap2, FLIR). The green channel recordings for each individual mouse were performed at the frame rate of 10 frames/s for 3.5-min trials. For the red channel, the recordings were performed at the frame rate of 10 frames/s at multiple focal planes by changing the tunable lens pulse-width modulation (PWM) control signal, capturing around 100 frames for each plane. Repetitive calcium and behavior recording was performed at the interval of 1 weeks for 4–5 months. The field of view was kept the same for an individual mouse during multiple recordings by taking the previous calcium recordings as reference and adjusting the focal plane of the miniscope by changing the PWM control signal from 0 to 1,023 as well as adjusting the X and Y offset.

For the *CaMKII-IRES-Cre; Tardbp^F/F^* mice, three *in vivo* calcium imaging recordings were made as baseline recordings for neurons before Tamoxifen induction. The mice were then fed with tamoxifen containing diet for around 18–21 days to induce the expression of Cre-recombinase dependent tdTomato. Repeated *in vivo* calcium imaging recordings during open field test were then performed for these mice once per week.

### 2.7 Neural data analysis

Each frame from of the miniscope CMOS sensor was filtered with a 3 × 3 median filter, and frames within each trial were motion corrected to compensate for *x*–*y* shifts caused by miniscope motion with a custom Fourier based image registration method. For the green channel, which contained the dynamic signal from GCaMP6f, neural footprints and traces were extracted for each trial using the constrained non-negative matrix factorization (CNMF) based method CaImAn (Giovannucci et al., 2019). For each session, comprised of 3 trials, neurons were registered (Sheintuch et al., 2017), and a session neural map was created by averaging the footprints of neurons on each trial they were detected. This map was used to estimate the activity of neurons in trials where they were not detected by CaImAn using an ROI based method (Barbera et al., 2016).

For the red channel, capturing the static fluorescence from tdTomato expressing neurons, the average image of the recordings for each focal plane was high-pass filtered and segmented to identify the soma of the tdTomato labelled neurons.

After registering the average image from the two channels on each session, single neurons were matched based on a minimum 50% overlap of their footprints.

### 2.8 Focal plane estimate

To estimate the effects of a change in the EWL control signal on the depth of the focal plane using the 45° depth of field target, we calculated the column-average pixel value from the time-average frame of the 100-frame trial at each focal depth, which reflects the 15 lp/mm vertical lines detected along the *x*-axis. The resulting curve is approximated as a Fourier series truncated at the first harmonic (tuned to the frequency of the line pairs, *w* = 66.67), multiplied by an exponential envelope curve, whose center parameter *b* indicated the estimated depth of the focal plane (Barbera et al., 2022):


$$a\;.\;e^{-{\left(\frac{x-b}{c}\right)}^{2}}.\;\left(A_{0}+A_{1}\cos\left(\omega x\right)+B_{1}\sin\left(\omega x\right)\right)$$

Based on the estimated focal depth for each control signal PWM, a linear model was fitted to approximate the relationship between control signal duty cycle and resulting focal plane depth.

## 3 Results

After testing the miniscope resolution on a 1951 USAF target (Figure 1B), we measured the focal depth range on a 45° depth of field target (Edmund Optics Depth of Field target DOF 5–15, 15 lp/mm). Since one of the applications of the dual color miniscope is the simultaneous recording of two spectrally distinct indicators (Figure 1C) by alternating light source and focal plane depth at each frame (Figure 1D), we quantified and compared the focal depth capabilities for both single-and dual-channel imaging: first we collected 100-frame trials from the green channel, each trial at a different focal depth, spanning the entire range of possible control signals for the EWL (0–100% PWM, spanning a 770 mm axial range) in 24 trials.

We then estimated the focal plane depth for each trial based on the center of the fitted envelope curve for the column-average pixel value of each trial’s average frame (Barbera et al., 2022), which represents the center of the focused 15 lp/mm vertical lines from the 45° depth of field target. With a similar procedure, in order to prove that a full focal plane shift is attained in alternating dual color imaging, we estimated the focal plane depth for each channel, in 20 trials with increasingly larger difference between the two channels’ focal planes (Figure 1E). By comparing the linear fit of the estimated focal plane for each EWL control signal (Figure 1F), we observed a similar slope for the single channel green (*y* = 6.57*x* + 91.73, *R*^2^ = 0.974), dual channel green (*y* = 8.31*x* + 376.54, *R*^2^ = 0.955), and dual channel red (*y* = 6.13*x* + 340.35, *R*^2^ = 0.908), indicating a similar response to the EWL control signal both in single-and dual-channel interleaved mode.

**Figure 2:**
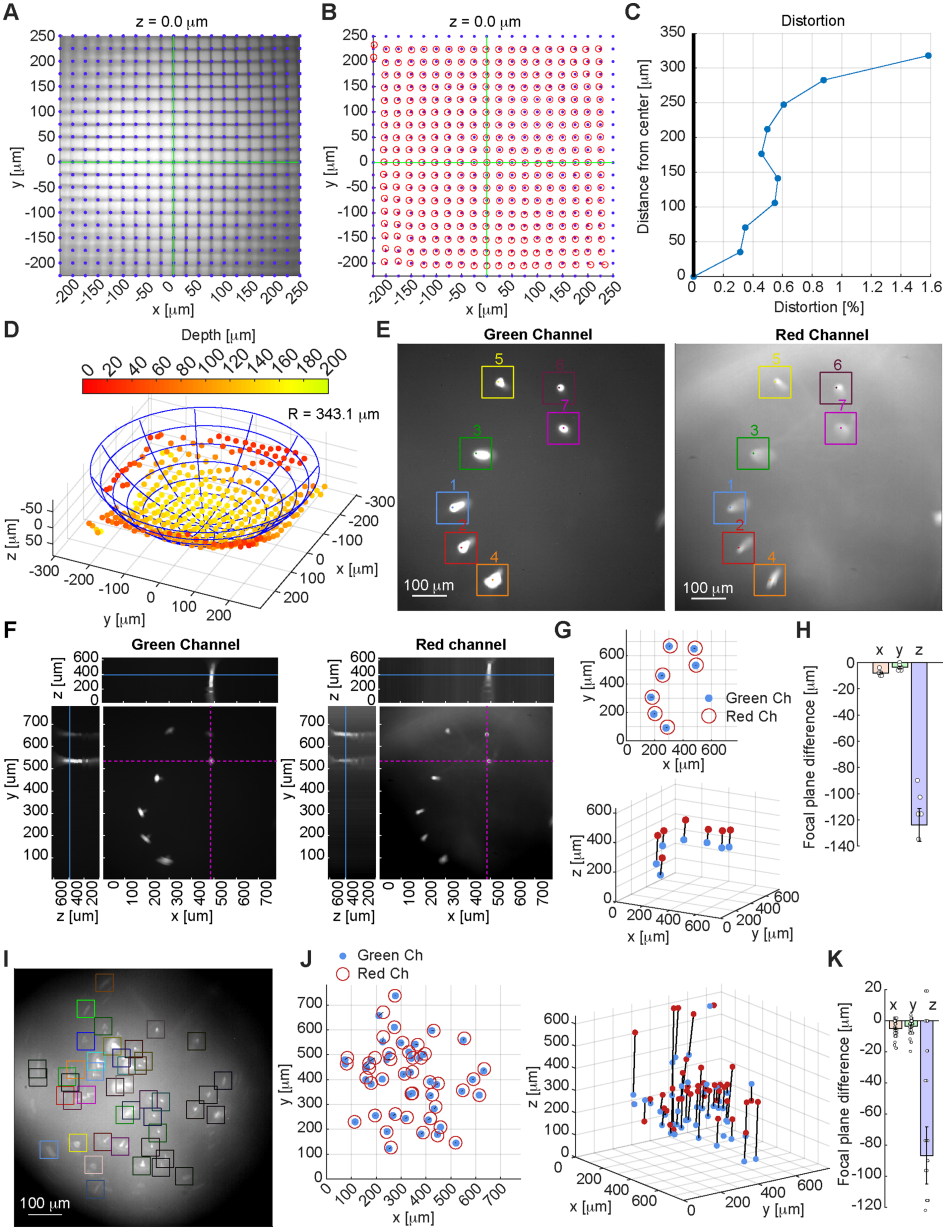
**(A)** Image of a 25 mm grid target calibration slide with overlayed the equally spaced 25 mm line intersections, which match closely the imaged intersections (*x* and *y* axes overlayed in green). Focal depth 0 is set as the depth where the largest number of intersections are in focus. **(B)** Overlay of the equally spaced 25 mm line intersections (blue dots) and detected intersections from the miniscope field of view (red circles). **(C)** Measurement of the system’s distortion. **(D)** Measurement of the Petzval field curvature: the optimal focal plane is calculated for each line intersection, and the fitted sphere radius and center are estimated by linear regression. **(E)** Sample image from green channel (left) and red channel (right) of a test slide with 15 mm diameter fluorescent beads: identified beads and peak brightness locations are highlighted and numbered. **(F)** Image of the green channel (left) and red channel (right) for the test slide shown in **E** at optimal focal depth for bead #7, showing *x*–*y*, *x*–*z*, and *y*–*z* planes at the *x*–*y* location of the representative bead (highlighted in purple dashed lines on the *x*–*y* plane). The focal depth of the image is marked with a solid blue line on the *x*–*z* and *y*–*z* planes (for the full axial scan see Supplementary Video S1). **(G)** Location of the peak value for each 15 mm fluorescent bead on the *x*–*y* plane (top) and *x*–*y*–*z* space (bottom) as detected on the green channel (blue dots), and red channel (red circles/dots). **(H)** Focal plane difference for the 15 mm fluorescent beads between green channel and red channel in the *x*, *y*, and *z* direction. **(I)** Sample image from a test slide with 4 mm diameter fluorescent beads (green channel). **(J)** Location of the peak value for each 4 mm fluorescent bead on the *x*–*y* plane (left) and *x*–*y*–*z* space (right) as detected on the green channel (blue dots), and red channel (red circles/dots). **(K)** Focal plane difference for the 4 mm fluorescent beads between green channel and red channel in the *x*, *y*, and *z* direction.

We then tested the miniscope on a 25 mm grid test slide (Figures 2A–D) using a 1 mm diameter GRIN lens (GrinTech NEM-100-25-10-860-S-0.5p), measuring reasonable levels of distortion, mostly introduced by the GRIN lens. We imaged the target at 36 different axial planes, by changing the focal plane though the EWL at 9.6 mm increments on the z direction. By detecting the optimal focal plane for each line intersection (Figure 2D), we estimated through linear regression a Petzval curvature radius of 343.1 mm.

The miniscope performance was also tested on 15 mm and 4 mm diameter fluorescent beads (Thermofisher F36909, Figures 2E–K and Supplementary Video S1): we measured limited lateral chromatic aberration (Figures 2H,K) and a more pronounced axial chromatic aberration (−123.7 mm ± 33.3 mm), for which focal plane adjustment by controlling the EWL could be necessary.

**Figure 3:**
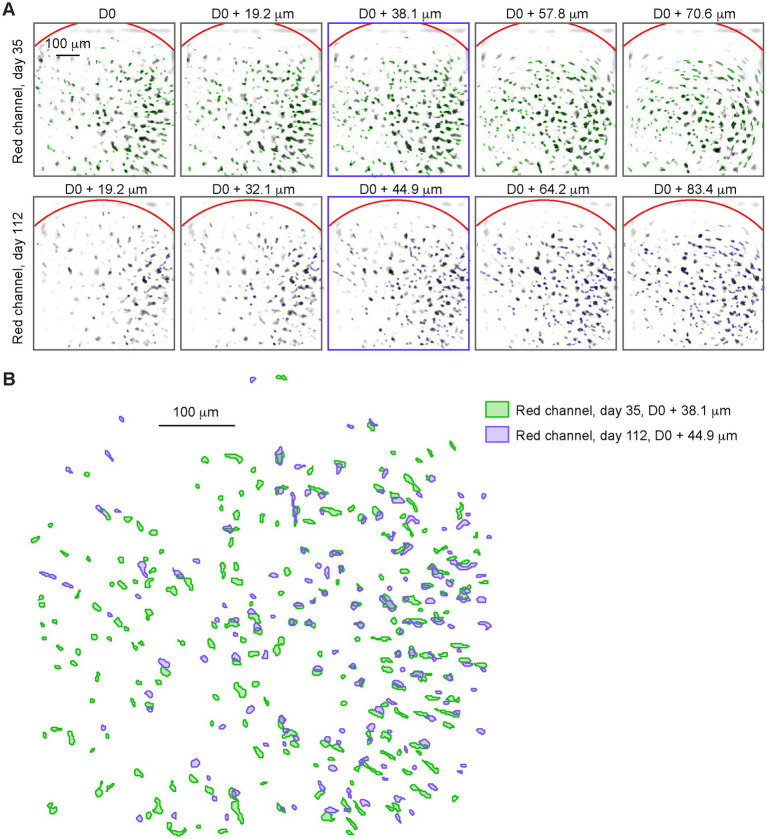
**(A)** Negative image of the trial-average red channel (565 nm LED) pixel value at 5 different focal depths (columns 1 through 5), on day 35 (top row) and on day 112 (bottom row). Reference depth D0 is set when mounting the baseplate. Neural maps detected at each depth are overlayed (green for day 35, blue for day 112). **(B)** Neural maps from the middle depth trial (marked with blue squares in **A**) on day 35 (green) and on day 112 (purple).

The miniscope was then tested with *in vivo* imaging in the mPFC during open field trials recorded at weekly intervals for several months. Two spectrally separate indicators were used to label distinct neuronal populations: before each open field session, 100 frames were recorded with only the 565 nm LED on (red channel), at 5 different focal planes (Figure 3A), to identify neurons labelled with tdTomato.

**Figure 4:**
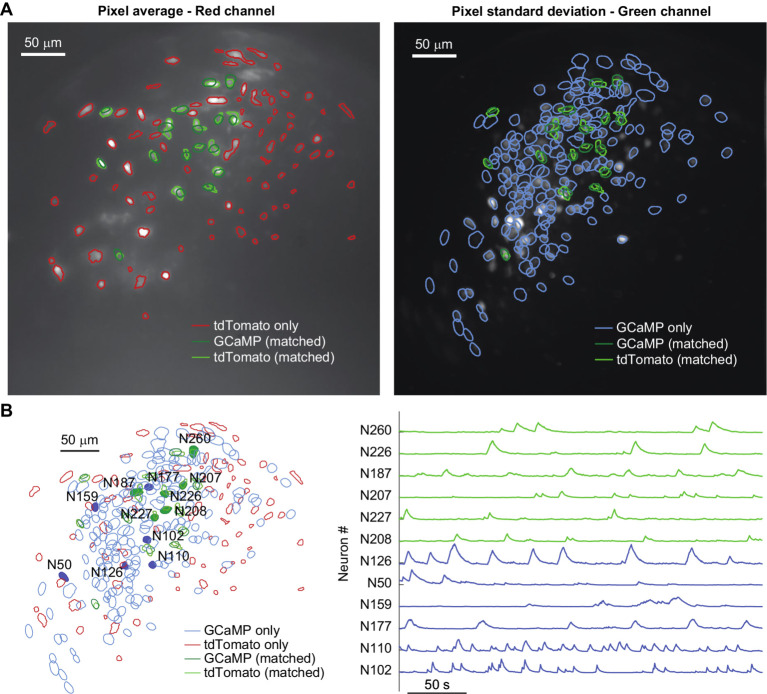
**(A)** Trial-average pixel value for a red-channel recording (left), and pixel standard deviation for a green-channel recording on the same day (right) of a Gad2-IRES-Cre mouse where the putative inhibitory neurons were labeled with AAV-CAG-DIO-NLS-tdTomato and all neurons were labeled with AAV-hSyn-GCaMP6f. Contours of neurons detected on each channel are shown, highlighting in green the ones that match on both channels (dark green represents GCaMP labelled neurons, and light green represents tdTomato labelled neurons). **(B)** Left: overlay of the neural maps identified in the red channel (red and light green contours) and green channel (blue and dark green contours); right: calcium traces for the representative neurons highlighted by solid colors in the neural map: traces in blue belong to neurons which are only detected in the green channel, while traces in green are from neurons detected in both channels (dark green represents GCaMP labelled neurons, and light green represents tdTomato labelled neurons).

Leveraging the ability to consistently adjust the focal plane through the electrowetting lens (EWL), it was possible to track neurons for up to 5 months (Figure 3B). On the same imaging days, 3.5-min open-field trials were recorded with the 470 nm LED on (green channel), recording the activity of GCaMP expressing neurons during free exploration (Figure 4A). This approach allowed us to identify specific subsets of neurons based on the overlap between the two channels and track the activity of distinct groups of neurons overtime.

**Figure 5:**
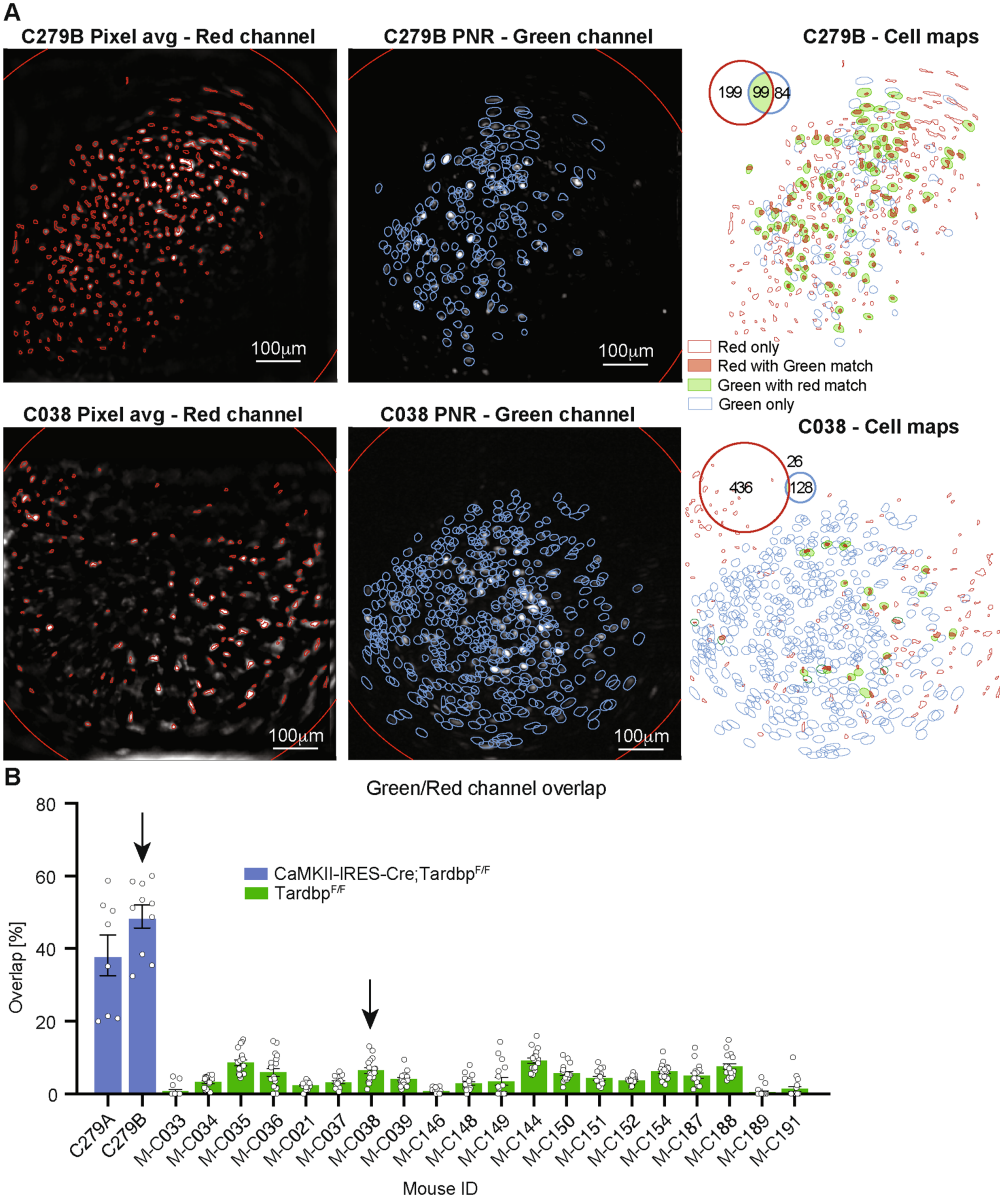
**(A)** Spatial maps showing average frame and detected tdTomato neurons’ contours for the red channel (left panel, detected neurons shown as red contours), frame peak-to-noise-ratio (PNR) and detected GCaMP neurons’ contours for the green channel (middle panel, detected neurons shown as blue contours), and overlay of the red-and green-channel neurons (left panel) for two representative mice (*CaMKII-IRES-Cre; Tardbp^F/F^* mouse on top row, and *Tardbp^F/F^* mouse on bottom row, respectively). Contours of neurons detected only in the red channel are shown in red, contours of neurons detected only in the green channel are shown in blue, and solid contours show neurons matched in both channel (red channel neurons with green channel match in solid red, green channel neurons with red channel match in solid green). Inset shows number of neurons detected on each channel and their overlap. **(B)** Barplot representing the percentage of neurons matched in both channels for different mice (single points represent individual sessions). Blue bars are from *CaMKII-IRES-Cre; Tardbp^F/F^* mice, labelling most excitatory neurons with AAV1-CAG-DIO-NLS-tdTomato and AAV1-CaMKII-GCaMP6f, while green bars are from retrograde labelling of layer 3 pyramidal neurons with AAVretro-hSyn-mCherry.

As proof-of-concept experiments, we first labeled out inhibitory neurons of Gad2-IRES-Cre mice with tdTomato and simultaneously monitored neural activity with GCaMP6f from both inhibitory and excitatory neurons in the mPFC (Figure 4B). We also labeled most excitatory neurons in the mPFC of Tamoxifen inducible *CaMKII-IRES-Cre; Tardbp^F/F^* mice with tdTomato, or employed retrograde labeling to mark layer III specific excitatory neurons with tdTomato (Wang and Sun, 2021). We recorded neural activity with GCaMP6f and compared the percentage of red-and green-channel overlapped neurons between the two scenarios. The overlap of the neuronal contours generated from the two video streams on each session was used to match neurons identified in the two channels (Figure 5A). As expected, for each session the overlap between red-and green-channel neurons was higher for *CaMKII-IRES-Cre; Tardbp^F/F^* mice (labelling most excitatory neurons with tdTomato) than for that of layer III pyramidal neurons (with retrograde labeling using AAVretro-hSyn-mCherry) (Figure 5B).

## 4 Discussion

Here, we presented a dual channel miniscope with variable focus capable of imaging distinct neuronal populations. The open-source design is adapted from Barbera et al. (2022), and one of the advantages of the custom miniscope platform is that it can be built with relatively inexpensive 3D-printed parts and off-the-shelf components. Another advantage of this approach is that the design is customizable, and it can be modified to fit specific needs and experimental requirements. For instance, by changing filters and light sources, one could extend the miniscope’s functionality to different indicators, or add optogenetics capabilities for simultaneous monitoring and manipulating neural activity of targeted groups of neurons.

We validated the miniscope functionality through *in vivo* imaging of two genetically distinct neuronal populations on the mouse mPFC during open field tests which spanned several months; when relying on the footprint of active neurons only, registration across many weeks and months can be challenging, since even during similar behaviors, different sets of neurons can be active at different time points. To improve the image registration accuracy in long-term imaging datasets, where focal plane drift and changes in brain structure limit the ability to register neurons across extended periods of time, the dual-color miniscope could be used in a configuration where one channel is dedicated to measuring neurons’ activity, and the other to statically label neurons and generate a stable neural map that can be used to provide a more precise registration of the tracked neurons.

For applications where the goal is to record neural activity from two neuronal populations expressing different GECIs, the dual-color miniscope can be used by alternating light source and focal plane every other frame. This is possible due to the fact that the switching time of EWLs are in the order of few milliseconds, which is a faster timescale compared to that of calcium dynamics and the typical frame rates for calcium imaging (10–60 fps). In this mode, however, the frame rate on each channel is reduced to half the original frame rate, resulting in a lower sampling frequency which may not be adequate to study neural processes or behaviors happening at faster timescales. Conversely, for higher frame rate imaging (>60 fps) and faster indicators—e.g., in the case of voltage sensitive dye imaging (VSDI), the switching time of the EWL could play a factor and should be carefully considered in the design of the imaging system.

## Data Availability

The datasets presented in this study can be found in online repositories. The names of the repository/repositories and accession number(s) can be found at: https://github.com/giovannibarbera/miniscope_v2.0.

## Footnotes

^1^ https://github.com/liang-bo/AutoStereota
